# Reconciling marine capture fisheries with the blue economy: Role of satellite-based advisory services

**DOI:** 10.1016/j.isci.2026.116421

**Published:** 2026-06-30

**Authors:** Dhanya M. Lal, Bhagyashree Dash, Sanjiba Kumar Baliarsingh, Alakes Samanta, N. Swetha, Sudheer Joseph, M. Nagaraja Kumar, T.M. Balakrishnan Nair

**Affiliations:** 1 Indian National Centre for Ocean Information Services, Ministry of Earth Sciences, Government of India, Hyderabad 500090, India

**Keywords:** Environmental science, Aquatic science

## Abstract

India’s blue economy policy envisages the responsible use of ocean resources for economic growth and sustainability. The Indian National Centre for Ocean Information Services (INCOIS) contributes to this vision by supporting the fishing industry through its Potential Fishing Zone (PFZ) advisory services. PFZ advisories help fishers reduce search time for fishing grounds, lower fuel costs, and encourage sustainable fishing. INCOIS thus plays a significant role in improving catch per unit effort and the livelihoods of fisher communities. This study traces the evolution of marine fishery advisory services for the Indian seas and examines the role and impact of PFZ advisories in India’s fisheries sector, with particular emphasis on their ecological, economic, and climate-related advantage over the years. For a more robust and sustainable blue economy, the results also highlight the need to combine traditional fishing knowledge with contemporary technological tools.

## Graphical abstract

**Figure:**
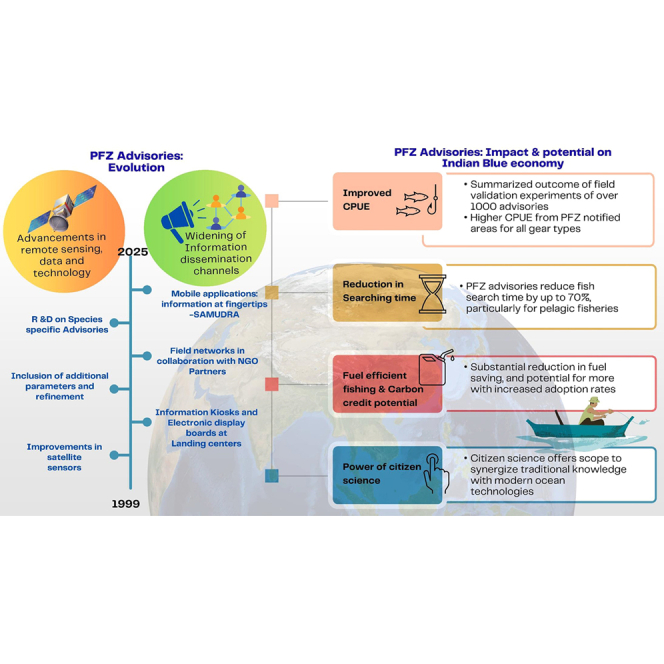

## Introduction

India has a large coastline of 11,098 km surrounded by the Bay of Bengal and the Arabian Sea.^1^^,^^2^ The Indian blue economy depends heavily on marine resources for food security, livelihoods, and coastal economic development. A major share of national income and exports comes from the fisheries sector, which also supports the livelihoods of millions of people, particularly coastal communities.^3^^,^^4^ In recent years, the sustainable use of ocean resources for economic growth, improved lifestyles, and ecological balance has been given more importance, along with its association with the blue economy^1^^,^^5^ The most difficult task here is to balance the need to preserve the marine ecosystem with the growing demand for oceanic resources. In addition, unregulated fishing methods, overfishing, and the effects of climatic variability are major issues that frequently threaten the sustainability of India’s maritime fisheries sector.

In India, the Potential Fishing Zone (PFZ) advisory services, an operational product derived from integrating modern technology with scientific-theoretical knowledge, have been proven to support marine fisheries with encouraging results. By integrating environmental data, together with satellite-derived information such as sea surface temperature (SST) (influencing fish movement and aggregation) and chlorophyll-*a* (chl-*a*) concentration (which serves as a proxy of primary productivity), a PFZ advisory provides information on the availability of productive fishing grounds that the fishermen can follow to find the fish.^6^^,^^7^ As evidenced by several published reports, following PFZ advisories for fishing reduce search time and fuel use, resulting in lower operational costs, higher profits, resilient communities, and reduced carbon footprints and CO_2_ emissions.^8^^,^^9^^,^^10^ PFZ advisories in turn discourage indiscriminate fishing by promoting planned operations and improving catch efficiency, thereby supporting sustainable resource utilization and contributing to climate change mitigation through reduced fuel consumption and lower emissions.^11^ Importantly, these advisories are not issued during the statutory fishing ban periods along the east and west coasts of India, which coincide with the peak breeding season of major fishery resources, thus reinforcing conservation objectives and protecting spawning stocks. Advisories are also excluded from designated marine protected areas and turtle nesting grounds, thereby supporting responsible and sustainable fishing practices. Moreover, the service contributes to multiple United Nations Sustainable Development Goals (SDGs) by linking ocean observation, satellite remote sensing, and operational oceanography with livelihood support, food production, and sustainable resource use.

By reviewing the results of nationwide validation experiments carried out over the years by various stakeholders,^11^^,^^12^^,^^13^^,^^14^ this study investigates the role and impact of PFZ advisories, including species-specific PFZ for yellowfin tuna and hilsa, in India’s fisheries sector, with a focus on their ecological, economic, and climate-related benefits. It demonstrates how the PFZ, based on satellite data, has improved the efficiency of fishing operations in the Arabian Sea and the Bay of Bengal while reducing carbon emissions by using less fuel and requiring less time to search for fish. This synthesis also presents case studies examining the gear-specific benefits of PFZ and the optimization of fishing effort under different PFZ adoption scenarios. Furthermore, emphasis is placed on the importance of technological advancements in supporting the country’s blue economy, preserving oceanic resources, and ensuring the long-term stability of coastal economies by integrating state-of-the-art marine information services with conventional fishing information.

## Methodological framework for literature review

A comprehensive search criterion was employed to identify published scientific literature on the development, validation studies and reported impacts of PFZ advisory services. The method involves three steps: first, collecting all available scientific publications and reports on PFZ for the Indian coast using Scopus, Google Scholar, and ResearchGate. Keywords such as “potential fishing zone” and “PFZ” were searched individually as well as in combination with words such as “Impacts of PFZ,” “Validation of PFZ,” “Economic profit of PFZ,” and “less fuel consumption and PFZ.” The second step was screening of articles based on PFZ development and impact assessment (duplicates were removed, and directly related articles were retained). The last step was extracting the summarized information from the selected articles. Overall, more than 70% (*n* = 26) of the publications were retrieved through Google Scholar. Of the remaining studies, 20% (*n* = 9) were sourced from ResearchGate, while less than 15% (*n* = 7) corresponded to gray literature (e.g., technical and institutional reports). Notably, nearly 50% (*n* = 19) of the total references were recent publications accessible only through the Scopus database. Therefore, consultation of multiple databases was necessary to ensure comprehensive and up-to-date literature coverage. To avoid missing any important information, the freely available platforms were used, as they were widely accessible and commonly used during the initial years of PFZ advisory development. After completing this process, various aspects of the PFZ were represented in graphs and tables using Excel and Matplotlib (Python v.3.13).

## Genesis of Potential Fishing Zone advisory services for India

The generation of PFZ advisories in India began during 1996–1997 at the National Remote Sensing Agency (NRSA), using SST data from National Oceanic and Atmospheric Administration - Advanced Very High Resolution Radiometer (NOAA-AVHRR). Owing to the limited spatial and temporal resolution of early satellite data, mapping the entire Indian coastline required data spanning 3 days, thereby limiting the issuance of advisories to twice a week. At first, the advisories were made using analogue base maps. Later, the establishment of the Indian National Centre for Ocean Information Service (INCOIS) in 1999 marked a turning point in transforming the PFZ initiative into an operational service supporting the Indian fishing community. In the next step, the launch of Indian Remote Sensing Satellite- P4 (Oceansat-1) in May 1999 opened the way for the Space Applications Centre (SAC) to finally develop a technique for generating PFZ advisories based on SST and chl-*a* data. In 2001, these methodologies were formally handed over to INCOIS, and in 2002, INCOIS began creating digital base maps using bathymetry, coastline, and landing centre information from the National Hydrographic Office, along with integration with GIS tools, and more frequently on alternate days.^15^ By 2007, SAC introduced methods to incorporate wind-borne movement into PFZ features. The year 2011 marked the initiation of daily PFZ advisory generation using multi-satellite SST and chl-*a* data, along with optimally interpolated SST from Group for High Resolution Sea Surface Temperature (GHRSST).^16^ By 2012, coverage was expanded to 587 landing centres from 267, and the next year, surface current magnitude and direction were incorporated, and the PFZ generation process was partially automated, reducing processing time. To increase accessibility, PFZ advisories were disseminated in regional languages, with PFZ maps enriched with sector details, coastline, landing centre information, and bathymetry contours, and made available daily.

**Figure 1:**
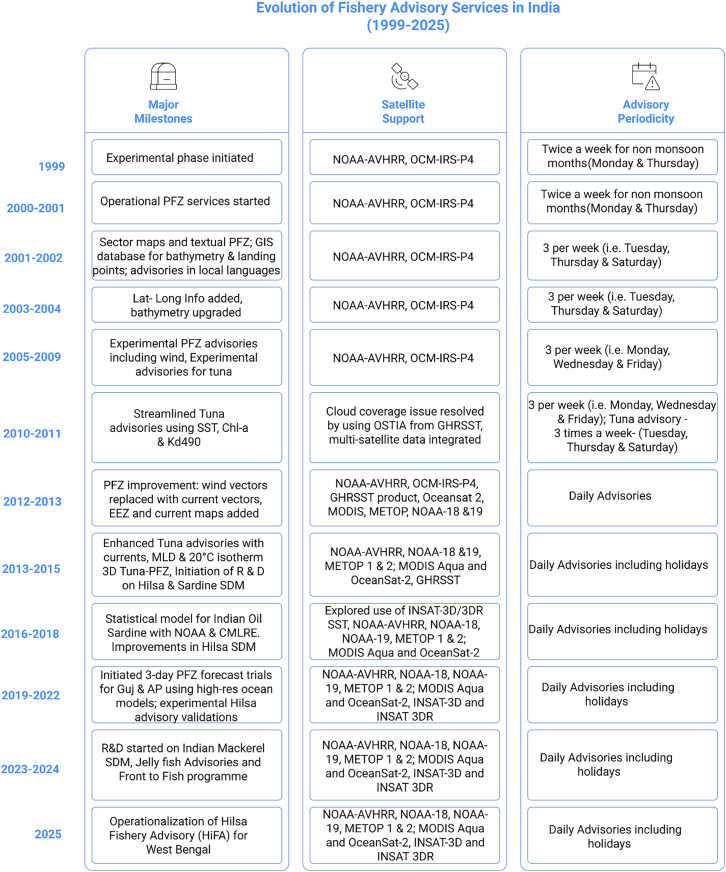
Scientific and operational milestones in the evolution of marine fisheries advisory services for the Indian seas

To expand the PFZ advisories, INCOIS began research to develop species-specific advisories based on the environmental optima and feeding habits of individual species. The species-specific PFZ initiative aimed to help fishers prepare appropriate craft and gear in advance. The first target species for this pioneering attempt was yellowfin tuna (*Thunnus albacares*), a highly sought-after fish with both ecological and economic significance in the Indian Ocean Tuna Commission (IOTC) region. The Marine Products Export Development Authority (MPEDA) initiated a scheme in 2006 to convert available vessels into tuna longliners as the foremost task to fill gaps due to the lack of a dedicated tuna fishing fleet in India. The next step was a request from MPEDA and industry stakeholders for the development of a Tuna advisory service similar to PFZ services. In response, INCOIS initiated research on the environmental preferences of yellowfin tuna, based on data on their feeding habits and target environmental conditions. Since information on tuna habitat and migration is limited, INCOIS initiated the SATTUNA project (Satellite Telemetry Studies on Migration Patterns of Tunas in Indian Seas) in collaboration with national fisheries institutes.^17^ Pop-up Satellite Archival Tags (PSATs) were used on yellowfin tuna to capture information on their vertical and horizontal movements, habitat preference, and environmental drivers. The combination of results from tagging research (species behavior data) with satellite oceanographic variables like SST, chl-*a*, and water clarity (Kd_490) provided a scientific basis for predicting tuna availability and abundance. With consideration of the yellowfin tuna’s migration pattern (diurnal) and it being a visual predator, the Kd_490 was taken as an essential predictor variable along with SST and chl-*a,* an improvement over the conventional PFZ perspective.^17^^,^^18^ Based on results from this project, INCOIS began providing experimental tuna-fishery advisories, which were subsequently improved and refined based on user input. Since November 2010, these advisories have been implemented and published via WebGIS and email to tuna longliner operators. Tuna fishery advisories are are disseminated only to registered users at present and are suspended during the uniform fishing ban seasons along both coasts of India along with the general PFZ advisory service. This approach serves as a precautionary measure to align science-based advisories with stock sustainability objectives, while promoting user accountability and balancing ecological considerations with the economic benefits of the tuna fishery. *Tenualosa ilisha* (hilsa shad) is among the most economically, ecologically, and culturally significant anadromous fish species in the Indo-Pacific. Nearly 90% of India’s total hilsa catch comes from the Hooghly estuary and neighboring waters of the northern Bay of Bengal, which are vital to the livelihoods of thousands of coastal fishermen. Yet, classical PFZ advisories based on SST and chl-*a* concentration are of limited utility for hilsa prediction. This is because hilsa distribution is strongly influenced by salinity gradients associated with its anadromous life cycle, as the species migrates between marine and freshwater systems for spawning. Therefore, salinity-driven habitat preferences play a more dominant role than temperature or chlorophyll during certain phases of its life cycle. To bridge this void and in response to persistent demand from the fishing community, INCOIS launched a focused research campaign to establish a hilsa-specific advisory service tailored to the West Bengal coast of India (upper north-western Bay of Bengal). A machine learning algorithm (XGBoost) was trained on geo-tagged catch data and satellite variables, including SST, salinity, and ocean currents, to identify high-potential hilsa fishing areas in the northern Bay of Bengal.^19^ The model was trained on georeferenced Catch Per Unit Effort (CPUE) data collected during the peak season, June to September, and its predictions were operationalized as a daily Hilsa Fishery Advisory (HiFA) service in April 2025, delivering targeted information to enhance the efficiency and sustainability of fishing in the northern Bay of Bengal. Importantly, the HiFA does not target spawning aggregations or estuarine breeding habitats; instead, advisories are generated exclusively for higher-salinity open marine waters, maintaining a 5–10 km coastal buffer and excluding peak breeding and trawl-ban periods. The service primarily targets post-spawning (spent) hilsa that migrate back to open marine waters after reproduction, thereby aligning the advisory framework with principles of sustainable fisheries management and conservation. The operationalization of the tuna and hilsa fishery advisory was a significant landmark in the PFZ advisory services system, combining traditional knowledge with advanced oceanographic techniques to improve scientific precision and user relevance. Figure 1 provides an overview of the major scientific and operational milestones in the evolution of PFZ advisory services for the Indian seas.

## Dissemination channels and stakeholder engagement

**Figure 2:**
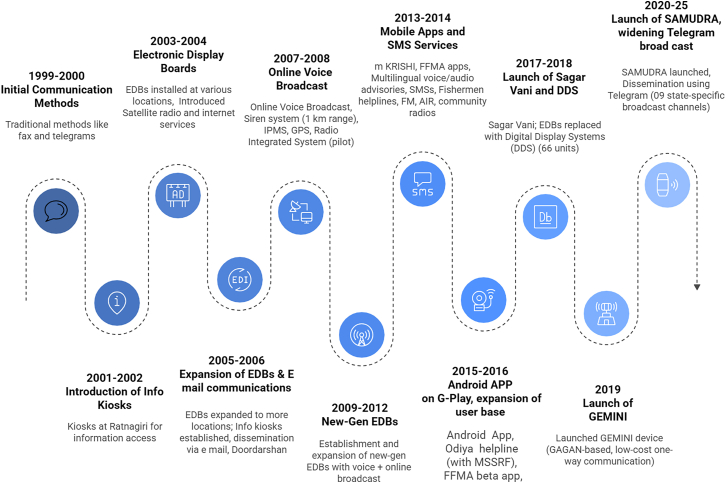
Evolution of marine fisheries advisory services dissemination systems in India

**Figure 3:**
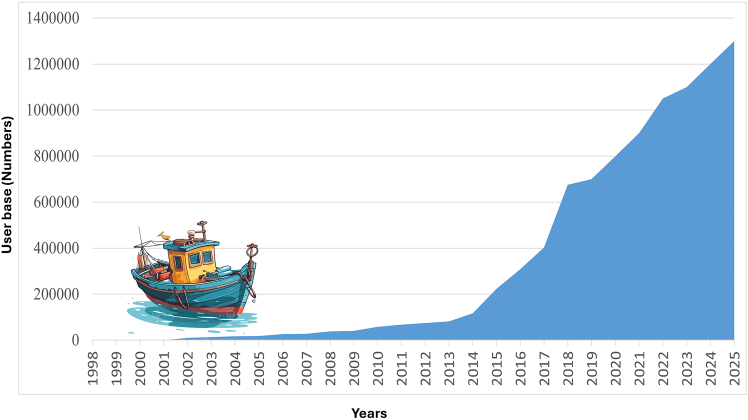
Year-wise expansion of the marine fisheries advisory services user base

From its inception and for more than a decade thereafter, the PFZ advisories were disseminated to a multitude of beneficiaries through several communication media, including Electronic Display Boards (EDBs), user-specific email, and fax messages to State Fisheries Departments, NGOs, and research organizations. INCOIS facilitated the installation of EDBs at major fishing jetties in the Indian coastal states, enabling fishermen to directly visualize the geolocations of fish availability at their convenience before venturing into the sea. The user-specific messages sent to different local agencies were subsequently delivered to the end users. Additionally, advisories are made available to all major ocean-sector stakeholders through the INCOIS web portal and email, while numeric data and spatial layers are provided to registered users via the INCOIS Web-GIS platform. Advisories specific to a region and time frame can also be delivered as per user-specific requests. In the initial phase, to enhance outreach among non-digital users and remote communities, INCOIS partnered with NGO-operated information kiosks and used platforms such as All India Radio, FM radio, local television channels, and NGOs to disseminate advisories, ensuring inclusive access. Over the last few years, INCOIS’s PFZ advisory services have delivered high accuracy and prompt service delivery, supported by satellite observation networks and advanced Information and Communication Technology (ICT) tools. Of these, the most important attempt was the pilot scale dissemination of advisories to distant offshore fishing vessels via satellites using the GEMINI (GAGAN Enabled Mariners Instrument for Navigation and Information) device. Besides, the Android phone-based SAMUDRA (Smart Access to Marine Users for ocean Data Resources and Advisories) app further expanded the accessibility by providing near real-time ocean data and fishery advisories to fishermen and other seagoers. In addition, access to these services is significantly expanded through partnerships with NGOs and their local dissemination networks, thereby facilitating last-mile delivery to coastal communities across India. Besides, INCOIS regularly organizes user interaction training and workshops, with participation from coastal schools and NGOs, to ensure effective service delivery. Figures 2 and 3 show the year-wise development of PFZ dissemination channels and the expansion of the fisher user base along India’s coastline, respectively.

## Impact assessment

PFZ advisories have significantly improved the efficiency, sustainability, and profitability of marine fishing operations across India. These advisories have led to a reduction in fish search time by 60%–70% for small pelagic fish shoals and 30%–40% for other commercially important species such as scombrids and carangids during ring seine operations.^20^^,^^21^ They have proven especially effective for pelagic gears like purse seines, gill nets, longlines and have also shown benefits for offshore and nearshore gears, regardless of depth.^22^

**Figure 4:**
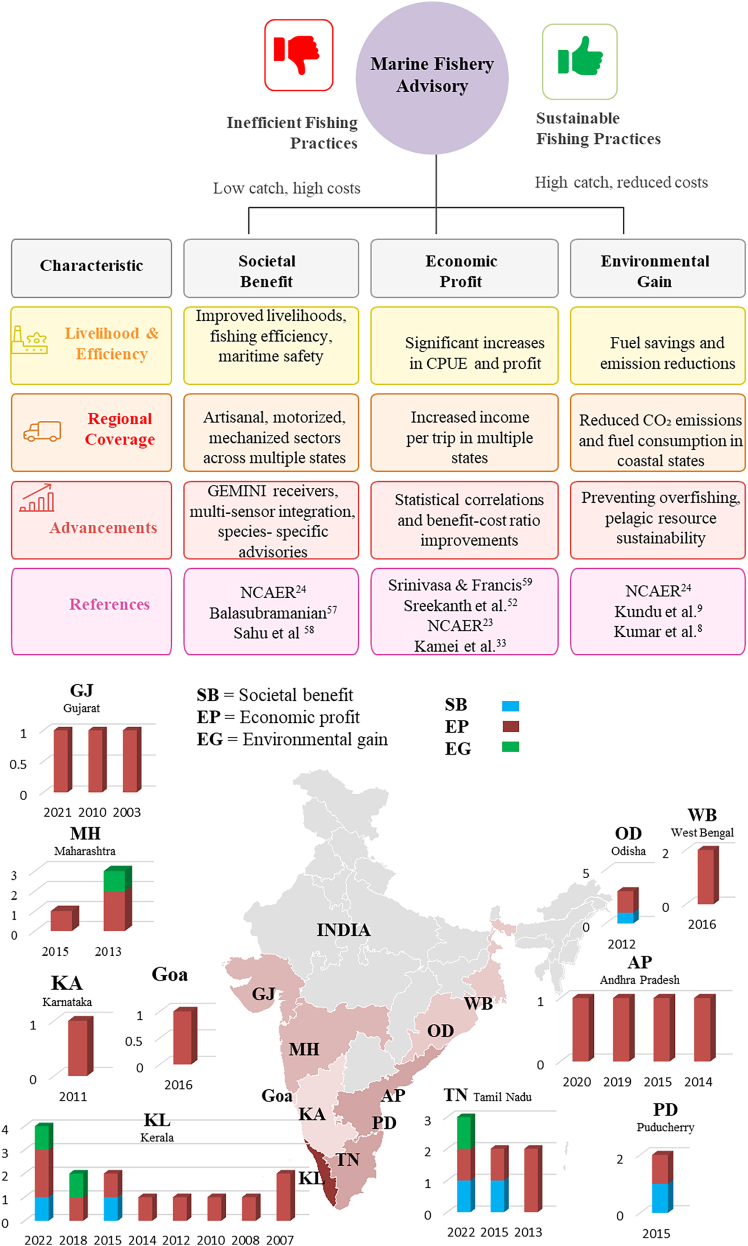
Schematic showing different aspects (socio-economic and environmental) of marine fisheries advisory services on the Indian coast The *y* axis on the bar plot represents the number of publications. References: Kumar et al.^8^; Kundu et al.^9^; NCAER^23^; NCAER^24^; Kamei et al.^25^; Sreekanth et al.^26^; Balasubramanian^27^; Sahu et al.^28^, and Srinivasa & Francis.^29^

In addition to direct benefit to fishing operations, PFZ advisories offer adequate financial and environmental advantages (Figure 4; Table S1). Assessments indicate an annual net capital benefit of Indian rupees 340–500 billion (3.74–5.51 billion USD) due to PFZ-guided fishing.^10^ A report on financial profit attributed to the use of PFZ advisories showed an average per-trip income increase of Indian rupees 0.018 million, totaling 19.2 million (211,522.47 USD) in yield from 1,079 fishing voyages.^23^ Additionally, reducing the time required for fishing operations from 3–5 days to 1–2 days resulted in lower fossil fuel consumption, fewer human hours spent fishing, and a smaller carbon footprint. For instance, it is noteworthy to mention that a reduction of 1 L of diesel consumption eschews ∼2.63 kg of CO_2_ emissions, which could translate to Indian rupees 2.84 trillion (31.29 billion USD) in carbon credit value over 25 years.^9^^,^^24^

PFZ validation experiments were conducted along both the east and west coasts of India (Table 1). As per an investigation in Raigad, Maharashtra, during the year 2013–2014, 15% adoption of PFZ advisories by the fishers saved 900,000 L of fuel, resulting in a cut of 2,412 tons of carbon emissions.^40^ Another study from the Kerala coast reported fuel savings of 21.5–1,293.5 L per ton of catch, corresponding to reduced carbon emissions of 0.06–3.45 tons.^8^

**Table 1:** Details of Potential Fishing Zone advisory validation experiment across Indian states, including period, fishing experiments, and gear types

State	Study	Period	No of advisories validated	No of Fishing experiments	Gears used
Gujarat	Solanki et al.^30^	2000	10	48	gill net
	Solanki et al.^31^	2000–2001	5	16	bottom trawl
	Solanki et al.^32^	1999–2002	81	81	bottom trawl
	Das et al.^33^	2008	19	19	bottom trawl
	Chavda et al.^21^	2007–2008	10	60	midwater trawl
Maharashtra	Thakare et al.^34^	2009–2007	46	428	purse seine
	Bhaware et al.^35^	2010–2012	107	107	bottom trawl
Goa	Sreekanth et al.^36^	2006–2012	290	290	purse seine
Karnataka	Deshpande et al.^37^	2008–2009	8	16	midwater trawl
		2009–2010	4	4	purse seine
Kerala	Nair^25^	2008–2012	126	126	ring seine
	Pillai and Nair^11^	2006–2010	55	55	ring seine
Tamil Nadu	Nammalwar et al.^13^	2007–2011	12	63	gill net
West Bengal	Dutta et al.^38^	2008–2011	182	182	gill net
	Giri et al.^39^	2010–2012	4	40	gill net
Andaman & Nicobar	George et al.^12^	2009–2012	50	50	gill net
			22	22	bottom trawl
			15	15	long line
Total			1,046	1,622	–

One of the leading Indian NGOs in the blue economy sector, the M. S. Swaminathan Research Foundation (MSSRF), also documented several pieces of evidence on the increasing utilization of PFZ advisories by fisherman communities, expanding acceptance and impacts on their lives.^14^^,^^41^^,^^42^ On the Indian coast, a few published reports also suggest scope for improving the service by considering certain peculiar features, such as the persistence of PFZs in the coastal-offshore region of the south-eastern Bay of Bengal and the prevalence of seasonal as well as depth-specific PFZs in the coastal waters of Andhra Pradesh and Kanyakumari.^43^^,^^44^^,^^45^^,^^46^ Furthermore, it has been observed that PFZ advisory generation based on near-real-time remote sensing data yields better catches, and therefore any significant delay in data reception and subsequent processing reduces the validity of PFZs, except when the water mass has not shifted, and physico-chemical-biological properties are not significantly changed.^20^ As reported, in most cases, a consistent high catch, with higher CPUE and species richness, was observed in PFZ zones than in non-PFZ regions.^39^^,^^47^^,^^48^ Recent research also suggests progressive trophic guild succession and a lagged response to PFZ formation.^48^ Such inferences can significantly strengthen the scientific basis of PFZ advisories by enabling the development of species-specific, probabilistic forecasts rather than generalized location-based guidance. Thus, they open avenues for making advisories more precise, ecologically informed, and temporally optimized. In the long-term, integrating trophic dynamics into advisory frameworks can support the evolution of PFZ services toward sustainable fishery advisory systems that balance catch efficiency with responsible resource use and conservation.

### Case study 1: Gear-specific benefit analysis of PFZ validation experiments over the Indian coastal states

Validation of PFZ advisories has been carried out along both the eastern and western coasts, as well as in various ecological settings, over the last few decades, using a variety of fishing crafts and gears. This case study investigates the fleet-specific advantages of PFZ advisories by considering all reported validation experiments across the Indian seas. The consolidated outcomes revealed a consistent increase in catch in PFZ-notified areas compared with non-notified regions, confirming the utility of satellite data for predicting fish availability (Table 2). Different metrics were used in the available literature for CPUE measurements, such as kg/h, kg/day, kg/trip, and kg/haul. To enable meaningful comparisons and synthesis across studies, appropriate conversions were applied to standardize the data.^49^^,^^50^^,^^51^^,^^52^ These conversions were carried out by considering the operational characteristics of each gear type and the approximate mean duration of fishing activity or number of days of fishing at sea(in multiday fishing trips), as reported in the respective studies and other reports. The result represents the CPUE of trawl and gill net catches in kg/h, seine catches in kg/haul, and the longline catches in kg/day.

**Table 2:** Summarized outcomes of gear-wise validation of Potential Fishing Zone advisories along the Indian coast

Gear type	No of Advisories validated	No. of fishing experiments	Mean CPUE-PFZ	Mean CPUE-NPFZ	CPUE unit	Major species
Gill net	258	383	36.89	13.58	kg/h	Indian Mackerel, Seer Fish, Hilsa, Tuna, Lesser sardines, Oil sardine, Carangids, Rabbit fish, Sword fish, Other Clupeids
Bottom trawl	234	245	298.34	195.65	kg/h	Prawns, Lizardfish, Shark, Ribbon Fish, Skate, Nemepterids, Cephalopods, Bullseye, Catfish, Other Perches
Mid-water trawl	18	76	41.60	19.00	kg/h	Clupeids, Carangids, Ribbon Fishes, Seer, Cephalopods, *Cynoglossus* spp., Prawns, Lizardfish
Purse seine	340	722	3260.53	1616.17	kg/haul	Indian Mackerel, Indian Oil Sardine, Horse Mackerel, Seer Fish, Coastal Tuna, Lesser Sardines, and Yellowfin Tuna
Ring seine	181	181	2621.22	603.27	kg/haul	Oil Sardine, Mackerel and Anchovies, Tuna, Seer Fish
Long line	15	15	969.33	518.32	kg/Day	Sharks, Bill Fishes, Marlins, Groupers

**Figure 5:**
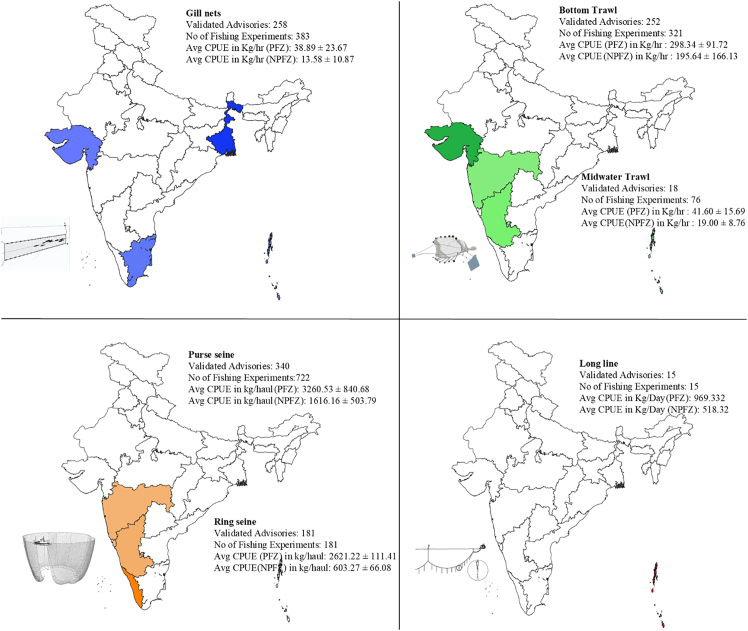
Gear-wise PFZ validation experiments across coastal states and the consolidated outcomes (top left: gill net, top right: trawl net; bottom left: seine net; bottom right: long line). “PFZ” indicates Potential Fishing Zones and “NPFZ” indicates non-Potential Fishing Zones (areas outside or away from a designated PFZ).

Several validation campaigns have been carried out by different agencies spanning almost all coastal states, with the largest number of experiments conducted in Gujarat and West Bengal. The predominant gear was gill nets, followed by trawls and purse seines across the locations (Table 1). A region-specific trend is observed in the use of gears for the validation experiments (Figure 5). Fish catch across gears varied between notified and non-notified regions under different fishing methodologies (Table 2). Maximum average catch rates with a mean CPUE value of 3,260.5 kg/haul were observed for purse seines in the PFZ region than in the non-PFZ regions, which was 1,616.2 kg/haul. This confirms the usefulness of PFZ advisories for better tracking of pelagic fishes such as mackerel, oil sardine, horse mackerel, seer fish, and coastal tuna. Species including sardines, mackerel, anchovies, and seer fish were more targeted by ring seiners and were mostly used in nearshore waters, and they also showed higher mean catches in PFZ regions (2,621.2 kg/haul) than in non-PFZ zones (603.3 kg/haul).

Bottom trawl, which mostly targets shrimps, ribbonfish, sharks, nemipterids, and lizardfish, showed a significant difference in CPUE: 195.6 kg/h in non-notified zones and 298.3 kg/h in notified regions. Artisanal fishers who predominantly use gill nets demonstrated a notably higher CPUE of 38.9 kg/h within PFZs compared to 13.6 kg/h outside, targeting a variety of pelagic fishes, including mackerel, seer fish, hilsa, and tuna. Mid-water trawls, which are reported to be less utilized than other gears, also showed significantly higher CPUE in PFZs (41.6 kg/h) than in the non-PFZ zone (19.0 kg/h). These generally catch clupeids, carangids, ribbonfish, and cephalopods. The longlines, which focus more on large fish such as sharks, billfishes, marlins, and groupers, also have higher CPUE (969.3 kg/day) in the PFZ region than in the non-PFZ region (518.3 kg/day).

Apart from these conventional methods, a few other validation exercises using non-conventional fishing approaches are also reported from different regions of the Indian coast. For instance, a validation exercise (2003–2005) along the Odisha coast using cast nets suggested notable advantages for fishing in the notified region, with a high CPUE of 40 kg/h in the PFZ, compared to 23 kg/h in the non-notified zone.^53^ Another survey-based assessment in Gilakaladindi village, located in Krishna District, Andhra Pradesh, highlighted the socio-economic benefits of PFZ advisory use.^14^ Dissemination of the PFZ advisories also helped increase catch rates and shift fishing practices. For example, gillnetting is now used more than bottom trawling due to higher capture rates of high-value targeted pelagic fish along PFZ lines, generating greater financial gains through improved fish quality, lower operational costs, and reduced fuel consumption. Collectively, the outcomes of experiments across the country strongly support the effectiveness of PFZ advisories across all gear types, enhancing both artisanal and mechanized fishing operations in India’s maritime states.

### Case study 2: Fishing effort optimization under various PFZ adoption scenarios

This case study assesses the usefulness of PFZ advisories for optimizing fishing effort to achieve sustainable fish catches and reduce emissions. The available literature on gear-wise fuel use (L/h) (Table 3), average fishing hours/day, and the percentage increase in CPUE when using PFZ advisories (Table 4) was compiled. For fishing fleets such as purse seine and ring seine, CPUE was reported as catch per haul, whereas for longline, it was reported as catch per day. To enable uniform comparison, all CPUE values were standardized to catch per hour, accounting for the average operational hours per day. Assuming that the same level of catch can be attained in proportionally reduced fishing time when CPUE increases, the required fishing hours under PFZ guidance were calculated as

$$H_{pfz}=H_{baseline}/\left(1+\Delta CPUE\right)$$

where H_pfz_ is the reduced fishing hours with PFZ use, H_baseline_ is the normal fishing hours/day,^49^^,^^50^^,^^51^^,^^52^ and ΔCPUE is the proportional gain (in percentage) in CPUE (while fishing in the PFZ area).

**Table 3:** Details of the fuel consumption for different fishing fleets available from the literature

Fleet type	Engine type	Average fuel consumption(L/h)	Major Fuel type	Horsepower (HP)	Reference
Gill netters	OBE	8–11	Diesel	60–70	Shibu and Hameed^51^
Single day trawl	IBE	9.5	Diesel	60–106	Devi et al.^52^
Multiday trawl (small)	IBE	19	Diesel	88–106	Devi et al.^52^
Multiday trawl (medium)	IBE	22.5	Diesel	88–400	Devi et al.^52^
Multiday trawl (large)	IBE	25	Diesel	99.27–411	Devi et al.^52^
Purse seiner	IBE	16	Kerosine	402	Kurup and Rajasree^49^
Ring seiner	OBE	13	Diesel	40	Kurup and Rajasree^49^
Ring seiner, carrier	OBE	8	Kerosine	25	Kurup and Rajasree^49^
Multiday long line (Gillnetter-cum-Longliner), IBM	IBE	8–18	Diesel	97–250	Vipin et al.^50^

**Table 4:** Input details used for calculating the fuel savings and CO_2_ emission reductions considering various potential fishing zone (PFZ) adoption scenarios

Gear type	No of advisories validated	No. of fishing experiments	Mean CPUE-PFZ (kg/h)	Mean CPUE-NPFZ (kg/h)	% increase in CPUE with PFZ use	Mean fuel consumption rate (L/h)	Hours of fishing per day (*H*_*baseline*_)	Fleet Size (*N*) as of 2010 (Vivekanandan^54^)	Fishing days per year (*D*)	CO_2_ emission per Litre of fuel consumed(kg/L)
Gill net	258	383	36.89	13.58	171.69	9.5	7	20,257	225	2.68
Bottom trawl	234	245	298.34	195.64	52.49	23.5	10	35,228	225	2.68
Purse seiner	340	722	465.79	230.88	101.74	15.5	7	2,200	225	2.68
Ring seiner	181	181	374.46	86.18	334.50	11.5	7	3,299	225	2.52
Long line	15	15	96.93	51.83	87.01	13	10	1,158	225	2.68

Source: Vivekanandan.^54^ Other information is linked to the sources listed in Tables 1 and 3.

Fuel saved per vessel per day was estimated as

$$F_{saved}=\left(H_{baseline}-H_{pfz}\right)*Fuel\;consumption\;rate$$

and scaled to annual fleet-level savings as

$$F_{annual}=F_{saved}*D*N*\alpha$$

where D = annual fishing days, N = fleet size, and α = adoption fraction of PFZ advisories (25%, 50%, and 75%). The corresponding CO_2_ savings were computed using fuel-specific emission factors (diesel = 2.68 kg CO_2_/L) and converted to tons of CO_2_. The inputs used for the calculation are listed in Table 4. The case study evaluates fishing hours, fuel consumption, and emission rates under different hypothetical adoption levels of PFZ services across various fleets.

**Figure 6:**
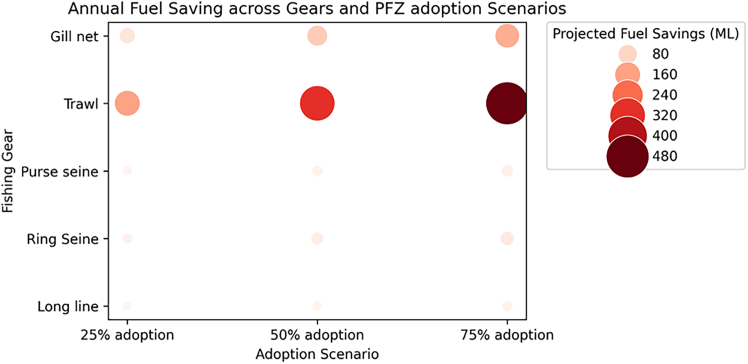
Bubble plot showing absolute annual fuel savings under various Potential Fishing Zone (PFZ) advisory adoption scenarios Projected fuel savings are expressed in Million Litres (ML).

**Figure 7:**
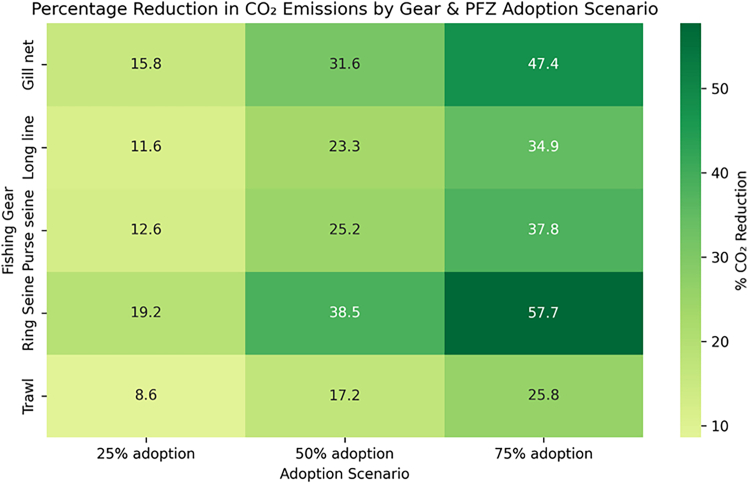
Heatmap showing fleet-specific % reduction in CO_2_ emissions (compared to No-PFZ-Guided Fishing) under various Potential Fishing Zone (PFZ) advisory adoption scenarios

The result suggested that the adoption of PFZ advisories could effectively help reduce annual fuel consumption for fishing (Figure 6) as well as CO_2_ emissions for all gear types (Figure 7). Under the 25% adoption scenario, where 25% of the fishing fleet follows the PFZ advisories and conducts fishing operations under optimization (normal man hours), the estimated annual CO_2_ reduction ranges from approximately 3.9 Million Litres (ML) for long lines to 160 ML for trawlers. Under the 50% adoption scenario, in which 50% of the fishing fleet follows the PFZ advisories, CO_2_ emissions are reduced by nearly half, with gill nets and ring seines achieving 31%–38% emission savings. In 75% adoption (if 75% of the fishing fleets are following the PFZ lines), ring seines demonstrate the highest relative emission savings (Compared to No PFZ-guided fishing with same type of fleet) of approximately 58%, followed by gill nets (∼47%), purse seines (∼38%), and long lines (∼35%). The larger emission reductions observed for ring seines, gill nets, and longlines suggest that these gears derive greater operational benefits from PFZ guidance, likely due to the enhanced efficiency of targeting pelagic fish aggregations. However, when it comes to total fuel consumption and emission reduction, the trawlers’ contribution is the highest, with a saving of approximately 481 ML annually at a 75% adoption scenario. Altogether, the higher the PFZ adoption levels, the greater the emission reductions and the lower the fuel consumption, underscoring the effectiveness of PFZ advisories as a potential sustainability tool. However, the scope of the current work was limited to the optimization of real fishing hours and does not specifically account for fuel savings arising from reduced search time. The reduced fishing hours were calculated from CPUE increases observed in original validation experiments, and the analysis predominantly explores the capability of PFZ advisories for sustainable resource utilization-an aspect not previously examined in detail elsewhere. In fact, incorporating search time reduction would likely further enhance the estimated fuel efficiency of fishing operations, but quantifying this component was not feasible with the available inputs. At the same time, it should be noted that these estimates are derived from hypothetical adoption scenarios. A potential concern is that higher levels of adoption could concentrate fishing effort within the notified areas, potentially leading to localized competition and a reduction in CPUE for individual fishers. On the other hand, from a conservation perspective, concerns regarding the overfishing of stocks also cannot be entirely ruled out. As the present analysis does not explicitly incorporate stock abundance or population dynamics, such aspects cannot be conclusively evaluated within the current framework. However, with improved ecological understanding and future advancements toward quantitative fishery advisories, these questions can be addressed more rigorously, and dedicated studies will become both feasible and meaningful.

## Improvement of marine fishery advisory services through integration of traditional knowledge with modern science: Citizen science approaches

The fishing communities hold generations of traditional knowledge that links to fish availability through the interpretation of moon phases, seabird activity, seawater coloration, and various other proxies. However, alterations in the climate and ocean environments, market fluctuations, and sea safety concerns limit the reliability and wide-scale applicability of the inherited practices.^26^^,^^55^ Climate change is altering fish distributions, phenology, and productivity, while overfishing, marine pollution, including microplastic and nurdle contamination, and rising global demand for seafood intensify pressures on already-stressed ecosystems.^56^^,^^57^ On the other hand, extreme events such as cyclones further alter fishing grounds but are often observed to trigger short productivity pulses.^58^

Modern scientific approaches and tools, such as satellite-based observations, vessel monitoring systems, other biogeochemical observations, and emerging technologies such as acoustic surveys and environmental DNA (eDNA), offer a more scientifically grounded understanding of the formation and evolution of fishing grounds. This not only enhances the predictive capability of potential fishing grounds but also increases the scale and reliability of insights that traditional knowledge alone cannot provide. Thus, integrating scientific data and insights with traditional knowledge and user feedback through citizen science approaches has great potential to improve conventional fishery advisories and develop species-specific advisories. This includes documenting the fishers’ traditional knowledge of fish aggregation, empowering them to log CPUE, bycatch, biodiversity observations, and pollution incidents, and integrating these insights to validate and improve the advisories. Such integration can operationally support ecosystem-based fisheries management by enabling spatially explicit fishing practices, improving monitoring of fishing pressure, bycatch, and biodiversity, and incorporating both environmental indicators and fisher-derived observations into adaptive, evidence-based decision-making. Thereby, citizen science provides the missing link by allowing communities to actively contribute and benefit from modern fishery advisory systems, thereby securing livelihoods and food security while preserving marine ecosystems.^59^

Thus, to transform fishery advisories into forecasts and species-specific predictions, the key requirement is geo-tagged fish catch data at large spatial scales. When such data are analyzed alongside collocated environmental data, it can provide valuable insights into the species’ habitat suitability and aid understanding of the evolution of the local fishing grounds. Recognizing this, INCOIS has adopted a citizen science approach through the development of a user-friendly mobile application, named “Fishery Information Source Hub (FISH),” enabling fishers to contribute to real-time data collection and submission using their smartphones. INCOIS, along with the collaborating institutes in the coastal states, has reached users through user interaction workshops, one-to-one demonstrations, multilingual leaflets, and onboard training during fishing cruises and has facilitated widespread adoption, leading to the successful installation of the app on over 1,000 devices in the initial phase. This initiative marks a shift from conventional PFZ experimental validations to participatory data collection, integrating traditional fisher knowledge with modern technology. The data generated through this approach will support ongoing R&D to develop species-specific advisories and understand food web dynamics in productive oceanic regions, combining satellite observations with AI/ML-driven modeling for more accurate, science-based fishery predictions.

## Challenges and limitations

Several challenges exist in refining the accuracy and improving the effective use of PFZ advisories, both from the source and user sides. On the user side, a lack of trust in adopting modern scientific information over the traditionally perceived know-how and low connectivity in offshore areas limit the timely receipt of advisories for planned fishing operations. These aspects often pose a challenge for real-time feedback, which is crucial for validating and improving the advisories. On the source side, the lack of seamless access to cloud-free satellite data, especially in the near-coastal area during the monsoon months, is a major challenge that hinders the generation of PFZ maps during these periods. Inadequate feedback, as mentioned earlier, is also a concern that hinders the science-based improvement in accuracy and reliability of the PFZ advisories. The key remedy to overcome challenges on both the source and user sides lies in coordinated approaches linking the stakeholders through citizen science. Approaches to fishery data collection using the FISH App can thus be considered promising, helping connect stakeholders without intermediaries and, to some extent, improving trust among users by fostering a sense of participation in R&D activities aimed at improving their livelihoods. The limitations arising from the lack of satellite observations could be overcome by developing and using advanced hyperspectral sensors, with consideration of airborne monitoring, to make fishery advisories more consistent and useful. In a nutshell, expanding geo-referenced catch reporting, integrating AI/ML techniques with advanced satellite and *in situ* observations, and maintaining continuous user engagement and two-way communication through workshops and onboard demonstrations will be the key strategies to overcome the current limitations in the reliability and wide-scale adoption of the fishery advisories. These efforts can bridge current gaps, enhance the accuracy of advisories, and transform them into dynamic forecasts and even species-specific predictions for sustainable resource use.

## Fishery advisory services and Sustainable Development Goals

The role of fishery advisory services in contributing to the UN SDGs and aligning with national priorities, particularly SDG 1 (No Poverty), SDG 2 (Zero Hunger), SDG 13 (Climate Action), and SDG 14 (Life Below Water), is elaborated in this section. SDG targets 1.2 and 1.5 aim to reduce the proportion of people living in poverty and decrease the vulnerability of poor communities to environmental and economic shocks. Small-scale fishing communities are economically vulnerable, and PFZ advisories reduce search time for fishing grounds, thereby lowering fuel use and operational costs. By reducing uncertainty in fishing operations, the advisories improve income stability and buffer fishers against fluctuations in resource availability and fuel prices.^27^^,^^28^ Thus, PFZ advisories act as a livelihood-stabilization and risk-reduction service, contributing to poverty alleviation and enhancing resilience to environmental variability.

SDG targets 2.3 and 2.4 aim to enhance the productivity and incomes of small-scale food producers and promote sustainable food production systems. PFZ advisories support these goals by improving catch efficiency rather than increasing fishing effort, thereby enhancing the CPUE. This increases the availability of marine protein in local markets without expanding fishing capacity or effort. Consequently, PFZ advisories strengthen nutritional security in coastal communities while maintaining sustainable harvesting practices.

The SDG targets 13.1 and 13.3 focus on strengthening resilience to climate-related hazards and improving climate awareness and adaptive capacity. By providing near-real-time oceanographic information, PFZ advisories help fishers adjust to environmental variability and shifting fish habitats. Reduced search time lowers fuel consumption and associated greenhouse-gas emissions, contributing to both climate adaptation and mitigation.^29^ Dissemination activities also enhance ocean and climate awareness, supporting informed decision-making among fishing communities.

SDG Target 14.2 emphasizes the sustainable management and protection of marine ecosystems. While the linkage is indirect, PFZ advisories may contribute to ecosystem-based fisheries management by reducing unnecessary search effort and associated fishing operations, which can potentially lower bycatch and unintended impacts on non-target species. However, it is important to note that in the absence of explicit catch management measures, the alignment of the advisories with SDG 14.2 remains limited and context-dependent. Nonetheless, withholding of advisories during closed seasons and within protected areas ensures their alignment with conservation objectives and responsible resource use. Along with the present contributions, and in response to the need for greater accuracy, reliability, and improved balance with sustainability considerations, the fishery advisory services are envisaged to improve further, integrating satellite and *in situ* data on multiple environmental variables alongside fishery data, dynamic ocean models, and AI-ML-based analytics to deliver more accurate and species-specific predictions. This continuous development is backed by multi-stakeholder collaboration at the grassroots level and aligns closely with the country’s blue economy vision to ensure a balance between the sustainable harnessing of ocean resources and inclusive economic growth.

Looking ahead, it is important to recognize that the current PFZ advisory framework is primarily designed to enhance fishing efficiency and does not, at present, incorporate mechanisms related to prescribing catch limits or regulating harvest levels for sustainable resource use. Given that this remains an evolving area of scientific and operational research, future advancements may progressively integrate fish stock characteristics, ecosystem indicators, and quantitative assessment approaches. Such developments, underpinned by robust scientific inputs, may in the long term enable the delivery of advisories that provide fishers with more reliable catch forecasts and, at the same time, support informed decision-making for effective fisheries management.

## Conclusion

The PFZ advisory services (including species-specific) have become an important contributor to India’s blue economy. In particular, the evolution of marine fishery advisory services in India demonstrates the transformative power of integrating satellite observations, ocean models, and fisher knowledge to enhance efficiency, sustainability, and resilience in the fisheries sector. By reducing fuel use, improving catch rates, and enabling species-specific advisories, these services contribute directly to India’s blue economy vision while supporting key UN Sustainable Development Goals. Strengthening participatory approaches, citizen science, balanced considerations with resource sustainability, and technological innovations will be crucial for ensuring long-term ecological balance, livelihood security, and climate-smart fisheries management. The salient outcomes of the present study carried out in the aforementioned context are as follows: (1) PFZ advisories reduce fish search time by up to 70%, particularly for pelagic fisheries; (2) adoption of PFZ-based fishing reduces fuel consumption and CO_2_ emissions across fishing fleets, and higher adoption levels can lead to even greater fuel savings, economic gains, and carbon credit benefits across all gear types; (3) total annual net economic benefits due to the use of PFZ advisories estimated to lie in the range of 3.7–5.44 billion USD with considerable gain in per trip income; (4) marine fisher communities benefit through enhanced efficiency, profitability, and sustainable practices; (5) integration of satellite observations with continuous fishery data is crucial for advancing PFZ advisory services; (6) citizen science offers scope to synergize traditional knowledge with modern ocean technologies; and (7) integrating sustainability and precautionary principles in the marine fishery advisories is essential to balance economic gains with long-term fishery resource sustainability.

### Limitations of the study

The present study is a structured review of the utility of PFZ advisories across different maritime regions and fishing gear types in India. The analysis and conclusions are primarily based on published literature and documented validation studies, which represent only a fraction of the actual PFZ users and field-level experiences. Therefore, the findings may not fully capture the complete scale of real-world adoption and impacts. Also, the estimates presented in one of the case studies are derived from hypothetical PFZ adoption scenarios and should be interpreted as indicative rather than precise. Similarly, concerns related to the overexploitation of fish stocks in a higher adoption scenario cannot be addressed adequately in the study, as the present analysis does not explicitly incorporate stock abundance, ecosystem dynamics, or population-level assessments. Nevertheless, the study provides a reliable synthesis of the available evidence on the operational, economic, and environmental benefits of satellite-based PFZ advisories and highlights the need for further advancements toward quantitative and ecosystem-informed fishery advisory systems.

## Data and code availability

All the data sources are well detailed in the manuscript.

## Acknowledgments

The authors gratefully acknowledge the contributions of different scientific organizations, researchers, technical staff, and fishermen who are directly or indirectly associated with INCOIS’s Potential Fishing Zone (PFZ) advisory services. The authors also sincerely thank the anonymous reviewers for their constructive comments and valuable suggestions, which have greatly contributed to improving the quality of the manuscript. The authors dedicate this manuscript in loving memory of Ms. Naga Swetha (one of the authors), whose contribution to generating and disseminating PFZ advisories bridged ocean science with the livelihoods of fishermen. This is 10.13039/501100004814INCOIS publication contribution number 714.

Funding: this work was carried out as a part of INCOIS’s in-house program Marine Ecological Applied Research.

## Author contributions

D.M.L., data curation, software, visualization, formal analysis, investigation, and writing – original draft; B.D., data curation, software, visualization, formal analysis, investigation, and writing – original draft; S.K.B., conceptualization, formal analysis, investigation, and writing – original draft; A.S., conceptualization, formal analysis, investigation, and writing – original draft; N.S., data curation, formal analysis, and writing – original draft; S.J., supervision, resources, and writing – review & editing; M.N.K., supervision and writing – review & editing; T.M.B.N., supervision, resources, and writing – review & editing. All authors read and approved the final manuscript.

## Declaration of interests

The authors declare that the research was conducted in the absence of any commercial or financial relationships that could be construed as a potential conflict of interest.
