# Supplementary material for: Reconciling marine capture fisheries with the blue economy: Role of satellite-based advisory services
Source: iScience. 2026 Jun 30;29(7):116421. doi: 10.1016/j.isci.2026.116421 (PMC13377866; doi:10.1016/j.isci.2026.116421)
Supplement: Document S1. Table S1 [file mmc1.pdf]

iScience, Volume *29*

Supplemental information

Reconciling marine capture fisheries with the blue economy: Role of satellite-based advisory services

Dhanya M. Lal, Bhagyashree Dash, Sanjiba Kumar Baliarsingh, Alakes Samanta, N. Swetha, Sudheer Joseph, M. Nagaraja Kumar, and T.M. Balakrishnan Nair

Supplementary Table 1 Impact assessment of Potential Fishing Zone (PFZ) advisories

Sl. No.	Title	Reference	Summary
1	Operational oceanographic services for the growth of blue economy in India	Kumar & Francis (2024)	Estimated economic benefit of INCOIS PFZ and OSF services to be ₹23,800 crores over 25 years.
2	Advances in Ocean State Forecasting and Marine Fishery Advisory Services for the Indian Ocean Region	Balakrishnan Nair et al. (2022)	Highlighting the world-class ocean information and advisory services by INCOIS to stakeholders across India and neighbouring regions for safe navigation and sustainable fishing.
3	Seasonality in recurrent Potential Fishing Zones along India's Southwest coast and its relationship to the prevailing hydrographic settings	Nair et al. (2022)	Recurrent PFZs, primarily located within the 50 m depth contour, vary seasonally and are most abundant during the Northeast Monsoon, offering insights to enhance fishing profitability.
4	Potential Fishing Zones Persistence along Southern Tamil Nadu: A Case Study	Ranjith et al. (2022)	An analysis of 260 PFZ advisory maps for Southern Tamil Nadu (2015–2017) revealed significant spatiotemporal variations, highlighting the effectiveness of INCOIS's satellite-based advisories in enhancing fish catch and providing valuable baseline data for fisheries resource correlation.
5	Oceansat3 Applications for Ocean State Forecast and Potential Fishing Zones Services	Balakrishnan Nair et al. (2021)	Suggested SST and Chlorophyll data from OCM3 could be used for PFZ generation
6	Evaluation of Fish Catch in Potential Fishing Zones (PFZs) off Veraval Coast, Gujarat	Chavda et al. (2021)	Revealed the effectiveness of PFZ advisories on the fish catches and future potential through integration with numerical and AI/ML-based systems to improve operational oceanographic services.
7	A technical assessment of the use of current geospatial technologies to derive marine fishery advisories in India and the Way Forward	Kundu et al. (2020)	Documented the evolution of geospatial technology in India’s Marine Fishery Advisories, led by INCOIS, ultimately aiming to enhance fisher profits, reduce emissions, and achieve SDG 14 for sustainable fisheries.
8	Economic Benefits of Dynamic Weather and Ocean Information and Advisory Services in India And Cost and Pricing of Customized Products and Services of	Venkatesan et al. (2015)	The growth rate of gross value added in marine fisheries GDP could rise from 3.9% to 7.8% annually. Diesel savings translate to carbon credits worth ₹36,200 crore per year, with a present value of ₹2.84 trillion over 25 years. PFZ and OSF services also enhance crew security, reduce manual labour, and

Sl. No.	Title	Reference	Summary
	ESSO NCMRWF & ESSO-INCOIS		allow more family time.
9	Trends in Depth-Wise Occurrence of Potential Fishing Zones in North Andhra Pradesh Coast	Edward et al. (2020)	Analysis of PFZ advisories (2012–2013) for the north Andhra Pradesh coast revealed that PFZ frequency increases with depth up to 200 m and towards higher latitudes from Kakinada to Kalingapatnam, likely influenced by salinity patterns, coastal orientation, offshore currents, and freshwater influx, with fewer PFZs observed in nearshore regions.
10	Modelling of marine ecosystem in regional scale for short term prediction of satellite-aided operational fishery advisories.	Chakraborty et al. (2019)	Attempted to overcome the satellite data reliance by using coupled physical-biogeochemical model data capable of simulating ocean features leading to PFZs. The use of model data provides an additional advantage towards transforming the existing service from advisories to forecast.
11	Seasonal occurrence of potential fishing zones along northern Andhra Pradesh coast	Edward et al. (2019)	Seasonal analysis of PFZ advisories for the North Andhra coast showed a nearshore concentration during summer, shifting to deeper and northern waters through the monsoon and post-monsoon, with frequent PFZ hits near Kakinada Bay, high intensity during monsoon in Visakhapatnam, and peak post-monsoon activity in Kalingapatnam.
12	Environmental benefits due to adoption of satellite-based fishery advisories.	Kumar et al. (2018)	PFZ advisories significantly reduce CO_2 emissions by minimizing search time during fishing; data from 69 ring seine operations off the Kerala coast showed average emissions of 0.161 t within PFZs versus 0.959 t outside, resulting in a total reduction of 55.052 t of CO_2.
13	Implementing a Spatial Model to Derive Potential Fishing Zones in the Northern Bay of Bengal Lying Adjacent to West Bengal Coast, India	Giri et al. (2016)	Developed a spatial model to derive PFZ in the northern BoB using satellite-derived SST and chlorophyll data, and found that the model-generated PFZs showed comparable CPUE values to INCOIS advisories, with significantly lower CPUE in non-PFZ areas, validating the model's effectiveness.

Sl. No.	Title	Reference	Summary
14	Correlation of phytoplankton biomass (chlorophyll-*a*) and nutrients with the Catch per Unit Effort in the PFZ forecast areas of the northern Bay of Bengal during simultaneous validation of the winter fishing season	Datta et al. (2016)	Demonstrated a strong positive correlation between CPUE and both phytoplankton biomass (chlorophyll-*a*) and key nutrients (DIP, DIN, silicate) in the northern BoB during the post-monsoon seasons of 2010–11 and 2011–12, with CPUE significantly higher in PFZ forecast zones (8.15 ± 6.08 kg/hour) compared to non-PFZ areas (3.51 ± 1.61 kg/hour).
15	Validations on satellite-based potential fishing zone advisories along Goa, south-west coast of India	Sreekanth et al. (2016)	Validation study of 290 purse-seine fishing operations off the Goa coast (2006–2012) showed that use of PFZ advisories halved scouting time and significantly increased CPUE (4188.6 kg/h vs. 1783.5 kg/h) and mean profit (₹2.72 lakhs vs. ₹0.86 lakh), with a 2.3-fold CPUE improvement and higher benefit-cost ratio (2.48 vs. 0.79), particularly benefiting pelagic species like Indian mackerel and oil sardine.
16	A Blue Ocean Innovation	Singh and Singh (2016)	In Raigad (Maharashtra), PFZ services led to a 30–40% increase in catch and 30% savings in fuel, while also providing fishers with information on wind, waves, weather, cyclone/tsunami warnings, and related updates.
17	Satellite data aid India's fishermen for better livelihood	Balasubramanian (2015)	PFZ advisory leads to a 30 to 70 per cent reduction in time spent catching fish, which, in turn, saves fuel and reduces carbon emissions. Over a decade, fishermen, who have taken technology into their hands, have witnessed a change after using the PFZ advisory.
18	Estimating the economic benefits of Investment in Monsoon Mission and High Performance Computing facilities	Venkatesan et al. (2020)	Using PFZ advisories, the fishermen get an average of Rs. 17,820 additional income per trip. In total, an additional income of Rs. 1.92 crore was generated from the 1,079 fishing expeditions made using PFZ Advisories.
19	Application of remote sensing in fisheries: role of potential fishing zone advisories	Kamei et al. (2013)	Study along the Mumbai coast using INCOIS PFZ advisories showed significantly higher mean CPUE in PFZ areas-30.7 kg/h (experimental) and 18.31 kg/h (fisher feedback)-compared to 18.7 kg/h and 5.23 kg/h, respectively, in non-PFZ areas, with PFZ regions also exhibiting better water quality, including higher dissolved oxygen and chlorophyll levels. High fish catch, low SST, and high Chl-a were observed in PFZ areas than in the non-PFZ areas.

Sl. No.	Title	Reference	Summary
20	Utility and impact of ESSO-INCOIS Services: reflections of fishers from Andhra Pradesh, Tamil Nadu, Kerala & Puducherry	Vedavalli and Velvizhi (2015)	
21	A Manual on the use of Potential Fishing Zone (PFZ) Forecast	Subramanian et al. (2014)	PFZ advisories help identify fish-congregation areas, reducing search time, fuel use, and fishing days, and are especially beneficial for pelagic and mid-water gears because they are based on surface parameters such as chlorophyll and SST.
22	On the persistent occurrence of potential fishing zones in the southeastern Arabian Sea.	Kripa et al. (2014)	Analyses indicate that the nearshore regions of the Arabian Sea off Kerala, with depths less than 50 m, occurred more in the PFZ advisory maps than in the mid continental shelf region and the continental slope. The northern regions of Kerala had persistent PFZ areas, especially between Calicut and Kannur. Relatively high river discharges in the area and the presence of high nutrient content in the discharges, due to high mangrove afforestation, are likely causes of the persistent occurrence of PFAs in these regions.
23	Potential fishing zone advisories and conversion from bottom trawling to gillnetting: role of MSSRF-INCOIS Partnership in Gilakaladindi village in Krishna district, Andhra Pradesh – a preliminary study	Vedavalli et al. (2014)	Regular availability and adoption of PFZ advisories in Gilakaladindi village, Andhra Pradesh, led to 50 fishing boats shifting from bottom trawling to gillnetting.
24	Applications of remote sensing in the validations of Potential Fishing Zones (PFZ) along the coast of North Tamil Nadu, India	Nammalwar et al. (2013)	North coast of Tamil Nadu from April 207 to March 2011 revealed fish catch in the PFZ is 3-4 times higher when compared to the non-PFZ area.
25	Impact on fishing patterns and life style changes of Kanyakumari fishermen due to fading potential fishing zones	Ravindran et al. (2013)	Temporal analysis of PFZ data (2003–2011) for the Kanyakumari coast, using clustering and data mining techniques, revealed that climate change and overfishing are driving pelagic fish to deeper, more distant waters, causing shifting PFZs and posing challenges for local fishermen reliant on these dynamic zones.

Sl. No.	Title	Reference	Summary
26	Validation of potential fishing zone forecast along the Ratnagiri coast of Maharashtra, India	Thakare et al. (2013)	A study conducted along the 288 km Mirkarwada coast showed that purse-seine vessels fishing in INCOIS-notified PFZs had a 43.90% higher catch rate ($P < 0.05$), a stronger catch-haul relationship ($Y = 1353.9X$ vs. $Y = 866.41X$), and a fishing success rate of 72.79%, resulting in increased CPUE, reduced fuel usage, and lower fishing hours compared to non-PFZ operations.
27	The Catch per Unit Efforts (CPUE) through Validations of Potential Fishing Zone Advisories along the Sindhudurg District Coast of Maharashtra State	Bhaware et al. (2013)	A study conducted from 2008 to 2012 at the Sindhudurg district revealed that trawl fishing within INCOIS-notified PFZs achieved higher CPUE than outside, with fishers saving up to 50% in fuel and time, and noted that PFZ advisories were especially beneficial for trawling up to 50 m depth and more effective for purse-seine operations.
28	Satellite-based potential fishing zone (PFZ) advisories - acceptance levels and benefits derived by the user community along the Kerala coast	Nair & Pillai (2012)	Long-term validation study (2003–2011) along the Kerala coast confirmed that INCOIS-generated PFZ advisories, based on satellite-derived chlorophyll and SST, significantly enhance CPUE and net profit, especially for key pelagic species, when fishing aligns closely with satellite data, as demonstrated by over 100 controlled experiments.
29	A comparative study on the feasibility of utilization of GHRSST product for operational generation of Potential Fishing Zone (PFZ) advisories in India	Kumar et al. (2012)	Comparative analysis found that the 5 km GHRSST-PP OSTIA SST product is suitable for generating PFZ advisories on cloudy or monsoon days, provided only large, persistent features are selected and dispersed features are avoided.
30	Validation of PFZ advisories–a case study along the Ganjam coast of Orissa, the east coast of India	Sahu et al. (2012a)	Validation of PFZ advisories along the Ganjam coast (2003–2005) showed higher CPUE, reduced search time, and lower fuel costs in PFZ areas compared to non-PFZ areas, confirming their effectiveness in improving fishing efficiency.
31	Socio-economic conditions of fisher folk vis-à-vis satellite technology in the coastal district of Ganjam, Odisha	Sahu et al. (2012b)	A socio-economic study of Ganjam fishers (2003–2005) revealed that poor weather, turtle nesting, trawler intrusion, and other challenges impacted livelihoods, and recommended infrastructure, modern gear, organized marketing, and better use of PFZ advisory services to improve CPUE and economic conditions.

Sl. No.	Title	Reference	Summary
32	Direct and Indirect Validation of Potential Fishing Zone Advisory off the Coast of Uttara Kannada, Karnataka	Deshpande et al. (2011)	Validation of off Karwar showed that catch was significantly higher in PFZ regions than in non-PFZ regions.
33	NCAER. (2010). Impact Assessment and Economic Benefits of Weather and Marine Services.	Venkatesan et al. (2010)	Total annual net economic benefits from the scientific identification of PFZs using satellite data are estimated to range from 34,000 to 50,000 crore.
34	Validation of potential fishing zones along Saurashtra coast, Gujarat	Das et al. (2010)	Reported High catch in the PFZ areas compared to non-PFZ locations.
35	Potential fishing zone (PFZ) advisories- Are they beneficial to the coastal fisherfolk? A case study along Kerala coast, South India	Pillai and Nair (2010)	A validation study (2006–2010) along the Kerala coast confirmed that INCOIS PFZ advisories significantly enhanced CPUE and economic returns—by 2 to 6 times in catch and 2 to 7 times in monetary benefits—for artisanal, motorized, and small mechanized fishers targeting pelagic species.
36	Benefits derived by the fishermen using potential fishing zone (PFZ) advisories	Kumar et al. (2008)	PFZ advisories enhanced fishing success to 92% (ring seining), 95% (gill netting), and 85% (bottom trawling), compared to 29%, 12%, and 0% in non-PFZ areas, with search time reduced by 30–70%.
37	Validation of Potential Fishing Zone (PFZ) Advisories (2006–2007)	Kumar et al. (2007)	PFZ advisories were highly beneficial for artisanal, motorised, and small mechanised pelagic fishers, reducing search time by 60–70% for oil sardine shoals and 30–40% for mackerel, anchovy, tuna, and carangids in ring seining, thereby saving fuel and effort.
38	Utilization of IRS P4 ocean colour data for potential fishing zone - A cost-benefit analysis	Nayak et al. (2003)	Average income increased from Rs. 38428 to Rs. 65315 for trawlers and from Rs. 1443 to Rs. 2742 for gill netters among PFZ users in Gujarat.

Balakrishnan Nair, T. M., Srinivas, K., Nagaraja Kumar, M., Harikumar, R., Nimit, K., Remya, P. G., Francis, P. A., & Sandhya, K. G. (2022). Advances in ocean state forecasting and marine fishery advisory services for the Indian ocean region. In *Social and Economic Impact of Earth Sciences* (201-227). Singapore: Springer Nature Singapore.

Balakrishnan Nair, T. M., Nimit, K., Lotliker, A. A., Modi, A., & Joseph, S. (2021). Oceansat3 Applications for Ocean State Forecast and Potential Fishing Zones Services. In *2021 IEEE International India Geoscience and Remote Sensing Symposium,* 90-93.

Balasubramanian, S. (2015). Satellite data aid India’s fishermen for better livelihood. *RURAL21.*

Bhaware, B.G., Kurhe, A. R., & Mane, U.H. (2013). The Catch per Unit Efforts (CPUE) through Validations of Potential Fishing Zone Advisories along Sindhudurg District Coast of Maharashtra State. *International Journal of Pharmaceutical and Biological Sciences Fundamentals*, *3*(01).

Chakraborty, K., Maity, S., Lotliker, A. A., Samanta, A., Ghosh, J., Masuluri, N. K., Swetha, N. & Bright, R. P. (2019). Modelling of marine ecosystem in regional scale for short term prediction of satellite-aided operational fishery advisories. *Journal of Operational Oceanography*, *12*(2), pp.157-175.

Chavda, V. M., Vaghela, D. T., Parmar, H. V., & Parmar, P. V. (2021). Evaluation of Fish Catch in Potential Fishing Zones (PFZs) off Veraval Coast, Gujarat. *Biological Forum – An International Journal 13*(3): 127-133.

Das, S., Madhu, V. R., Sreejith, P. T., Jethva, J. K., & Meenakumari, B. (2010). Validation of potential fishing zones along Saurashtra coast, Gujarat. In *Coastal Fishery Resources of India: Conservation and Sustainable Utilization*, 360-369.

Deshpande, S. P., Radhakrishnan, K. V., & Bhat, U. G. (2011). Direct and indirect validation of potential fishing zone advisory off the coast of Uttara Kannada, Karnataka. *Journal of the Indian Society of Remote Sensing*, *39*(4), 547-554.

Dutta, S., Chanda, A., Akhand, A., & Hazra, S. (2016). Correlation of phytoplankton biomass (Chlorophyll-a) and nutrients with the catch per unit effort in the PFZ forecast areas of Northern Bay of Bengal during simultaneous validation of winter fishing season. *Turkish Journal of Fisheries and Aquatic Sciences*, *16*(4), 767-777.

Edward, L. L., Satish Kumar, M., Suresh Kumar, P., & Ghosh, S. (2020). Trends in Depth-Wise Occurrence of Potential Fishing Zones in North Andhra Pradesh Coast. *National Academy Science Letters*, *43*(1), 49-52.

Edward, L., Rao, M. V., Uma Mahesh, V., Muktha, M., & Ghosh, S. (2019). Seasonal occurrence of potential fishing zones along northern Andhra Pradesh coast. *Indian Journal of Geo-Marine Sciences*, *48*(2), 228-232.

Giri, S., Manna, S., Chanda, A., Chowdhury, A., Mukhopadhyay, A., Chakraborty, S., & Hazra, S. (2016). Implementing a spatial model to derive potential fishing zones in the Northern Bay of Bengal lying adjacent to West Bengal Coast, India. *Journal of the Indian Society of Remote Sensing*, *44*(1), 59-66.

Kamei, G., Felix, J. F., Shenoy, L., Shukla, S. P., & Devi, H. M. (2013). Application of remote sensing in fisheries: role of potential fishing zone advisories. In *Geospatial Technologies and Climate Change,* pp. 175-186. Cham: Springer International Publishing.

Kripa, V., Mohamed, K. S., Prema, D., Mohan, A., & Abhilash, K. S. (2014). Persistent occurrence of potential fishing zones in the southeastern Arabian Sea. *Indian Journal of Geo-Marine Sciences*, *43*(5), 737-745.

Kumar, M. N., Kumar, T. S., Swetha, N. N., Maity, S., & Jemima, U. (2012). A comparative study on feasibility of utilization of GHRSST product for operational generation of potential fishing zone (PFZ) advisories in India. *International Journal of Earth Sciences and Engineering*, *5*, 240-246.

Kumar, M. N., Nair, P., Pillai, V. N., Kumar, T. S. (2018). Environmental benefits due to adoption of satellite-based fishery advisories. *Fishery Technology*. 55(2):100–103.

Kumar, T. S., & Francis, P. A. (2024). Operational oceanographic services for the growth of blue economy in India. *Current Science*, *126*(2), 208.

Kumar, T. S., Kumar, M. N., & Nayak, S. (2008). Benefits derived by the fisherman using Potential Fishing Zone (PFZ) advisories. In *Remote sensing of inland, coastal, and oceanic waters. 7150*, 127-138.

Kumar, T. S., Kumar, M. N., Padmaja, N. S., Nayak, S., Pillai, V. N., Reddy, K. G., Subramanian, S., Kumar, N. A., Nammalwar, P., Rajan, U. H. and Radhakrishnan, K. V. (2007). Validation of Potential Fishing Zone (PFZ) Advisories (2006–2007). *Indian National Centre for Ocean Information Services, Ministry of Earth Sciences, Govt. of India*.

Kundu, S. K., Santhanam, H., & Srikanth, R. (2020). A technical assessment of the use of current geospatial technologies to derive marine fishery advisories in India and the Way Forward. In *Asian Conference on Remote Sensing*.

Nair, P. G., & Pillai, V. N. (2012). Satellite based potential fishing zone (PFZ) advisories-acceptance levels and benefits derived by the user community along the Kerala coast. *Indian Journal of Fisheries*, *59*(2), 69-74.

Nair, P. G., Joseph, S., Pillai, N., & Abdulla, M. H. A. (2022). Seasonality in recurrent Potential Fishing Zones along India's Southwest coast and its relationship to the prevailing hydrographic settings. *Regional Studies in Marine Science*, *51*, 102191.

Nammalwar, P., Satheesh, S., & Ramesh, R. (2013). Applications of remote sensing in the validations of potential fishing zones (PFZ) along the coast of North Tamil Nadu, India. *Indian Journal of Geo-Marine Sciences*, *42*(3), 283-292.

Nayak, S., Kumar, T. S., & Kumar, M. N. (2007). Satellite based fishery service in India. *The Full Picture*, 256-257.

Venkatesan, R., Munjal, P., Sharma, A., & Meattle, C. (2015). Economic Benefits of Dynamic Weather and Ocean Information and Advisory Services in India and Cost and Pricing of Customized products and Services of ESSO-NCMRWF & ESSO-INCOIS. *National Council of Applied Economic Research (NCAER)*, *New Delhi, India. 137*.

Venkatesan, R., & Joshi, L (2010). Impact assessment and economic benefits of weather and marine services. *National Council of Applied Economic Research (NCAER)*, *New Delhi, India. 105*.

Venkatesan, R., Munjal, P., Sharma, A., Ali, S., & Pratap, D. (2020). Estimating the economic benefits of investment in Monsoon Mission and high performance computing facilities. *National Council of Applied Economic Research, New Delhi, India. 76.*

Pillai, V. N., & Nair, P. G. (2010). Potential fishing zone (PFZ) advisories-Are they beneficial to the coastal fisherfolk? A case study along Kerala coast, South India. In *Biological Forum* (Vol. 2, No. 2, pp. 46-55).

Ranjith, L., Edward, L. L., Kalidas, C., Prabu, D. L., Babu, A. M., Karuppasamy, K., & Zacharia, P. U. (2022). Potential Fishing Zones Persistence along Southern Tamil Nadu: A Case Study. *Indian Journal of Ecology*, *49*(6), 2306-2311.

Ravindran, N., Samraj, S. A., & Kavitha, C. (2013). Impact on fishing patterns and life style changes of Kanyakumari fishermen due to fading potential fishing zones. In *International Conference on Green High Performance Computing*.

Sahu, K. C., Baliarsingh, S. K., Srichandan, S., Lotliker, A. A., & Kumar, T. S. (2012b). Socio-economic conditions of fisher folk vis-à-vis satellite technology in coastal district of Ganjam. Odisha, *Review of Research*, *1*(4)1-6.

Sahu, K. C., Baliarsingh, S. K., Srichandan, S., Lotliker, A., & Kumar, T. S. (2012a). Validation of PFZ advisories–a case study along Ganjam coast of Orissa, East coast of India. *Indian Streams Research Journal, 1*(12), 1-11

Singh, V. V., Singh, D. P. (2016). mKRISHI® Fisheries – A Blue Ocean Innovation. Marine Fisheries Information Service; Technical and Extension Series (230) ISSN 0254-380 X. 3-6.

Sreekanth, G. B., Subramaniam, S., Manju Lekshmi, N., Madhu, V. R., Passta, M. F., & Singh, N. P. (2016). Validations on satellite based potential fishing zone advisories along Goa, south-west coast of India. *Indian Journal of Fisheries, 63*(1), 8-15.

Subramanian, S., Sreekanth, G. B., Manjulekshmi, N., Singh, N. P., Kolwalkar, J., Patil, T., & Fernandes, P. M. (2014). A Manual on the use of Potential Fishing Zone (PFZ) Forecast. *Technical bulletin*, (40), 27.

Thakare, A. U., Madhukar, S. M., Pai, R., & Sawant, M. S. (2013). Validation of potential fishing zone forecast along the Ratnagiri coast of Maharashtra, India. *Ecology, Environment, and Conservation, 19* (3), 853-858

Vedavalli, L., Velvizhi, S. (2015). Utility and impact of ESSOINCOIS Services: reflections of fishers from Andhra Pradesh, Tamil Nadu, Kerala & Puducherry. Chennai: M S Swaminathan Research Foundation (MSSRF). MSSRF/ RR/15/38, PB57.

Vedavalli, L., Velvizhi, S., Suvitha, D., Rao, N. V., Rao, D. S., Dhanraj, K., Maity, S. (2014). Potential fishing zone advisories and conversion from bottom trawling to gillnetting: role of MSSRF-INCOIS Partnership in Gilakaladindi village in Krishna district, Andhra Pradesh – a preliminary study. Chennai: M S Swaminathan Research Foundation. (MSSRF). MSSRF/RR/ 14/37,PB:48.
